# Validity of self-reported criminal justice system involvement in substance abusing women at five-year follow-up

**DOI:** 10.1186/1471-244X-8-2

**Published:** 2008-01-07

**Authors:** Irene Jansson, Morten Hesse, Mats Fridell

**Affiliations:** 1 Lunden Compulsory Care Home, Lund, Sweden; 2 Centre for Alcohol and Drug Research, Købmagergade 26E, 1150 Copenhagen C, Germany; 3 Department of Psychology, Lund University, Sweden

## Abstract

**Background:**

Few studies have compared self-reported criminal behaviour with high-quality databases of criminal offences and judicial sanctions. Self-reported problems from drug abusers are generally believed to be valid. We assessed the validity of self-reported theft, drug offences and prison sentences from a five-year follow-up of female substance abusers who were originally treated in a compulsory care unit in Lund, run by the Swedish Board of Institutional Care.

**Methods:**

Data from a total of 106 of a consecutive sample of 132 women inter-viewed in a five-year follow-up. All were thoroughly assessed for somatic complaints, psychiatric and psychological problems, background factors with standardized instruments. Data over the five years were linked to official records of judicial sanctions, retrieved from The National Council for Crime Prevention, Stockholm, Sweden. Register data have a full cover for the whole cohort. The current data base contain full data back to 1975 up to 2004.

**Results:**

Agreement was assessed for each year, as well as for the total period. Statistical control was performed for other types of crimes and prison. Although statistically significant, agreement was modest, and in contrast to previous studies, patients under-reported violence charges.

**Conclusion:**

The findings suggest that self-reports of criminal behaviour from women can be used with some caution, and that the validity of self-report may vary between types of criminal justice system involvement.

## Background

Relatively little is known about the validity of self-reported criminal behaviour in drug abusers. In a review from 1998, Darke found that the literature on the validity of self-reported crime was quite limited, but reported overall that for crime as well as for drug use patients were more likely to over-report than under-report, and generally reported that over-reporting and under-reporting cancelled out each other [1]. Studies from the 70ties and 80ties from the USA have indicated that drug abusers tend to report more crimes than is found in official records, a finding that may indicate incomplete data in the official databases [1,2]. Many early studies have been conducted on treatment populations, either at admission or during the course of treatment [1].

In a more recent study, Crisanti and colleagues found a fairly high level of agreement over a three-month recall period after treatment, although with a higher level of false negatives than false positives [3]. In a study by our group with a sample of voluntarily treated substance abusers of both genders from a detoxification and short-term rehabilitation unit, we found that agreement for incarceration was good, but somewhat lower for drug-related offences [4].

No studies have yet directly assessed the validity of self-reported criminal behaviour for substance abusing women. Women are generally a minority in substance abuse treatment samples and criminal justice samples, and findings may differ for women in several areas.

In the criminal penal system in Sweden, women constitute around 20% of all convicted persons. The proportion of all women registered in the Swedish criminal justice database, who are charged with drug offences are in 2005 approximately 15%, a figure that has remained very stable over the past 10 years [5]. Selling sex is not illegal in Sweden; therefore prostitution is not a ground for legal problems for women.

In general, amphetamines are among the most commonly used drugs among drug abusers in Sweden, between 35 to 40 %, and have been so since the 70ties [6,7].

The purpose of the present study was to assess the convergent validity of self-reported and criminal justice database information on criminal problems in women drug users.

## Methods

### Setting

The setting was a 21-bed inpatient compulsory care residential care unit, Lunden, in Lund Sweden. The institution has 12 beds for adults and 9 for youth. The unit staff includes psychologists, psychiatrist, nurses, social workers, treatment attendants, and administration.

Women are treated under the Law on Compulsory Care for Substance Abusers (LVM, act 1988:870) or The Care of Young Persons Act (LVU, act 1990:52).

According to the LVU, "A care order is to be issued, if the young person exposes his health or development to a palpable risk of injury through the abuse of addictive substances, criminal activities, or some other socially degrading behaviour." (LVU, act 1990:52, section 3). Youth can also be taken into care under the LVU due to neglect or chaotic circumstances in the family.

Under Section 4 of the LVM, a court can order compulsory care for a person whose health is deemed to be at risk, or who may be placing others at risk, and who is considered to need assistance in order to discontinue substance use. The LVM and LVU acts are unrelated to penal code and laws of psychiatric care.

Patients are usually reported to courts by social welfare, or, more rarely, police, their family members or general practitioner. Within 8 days after report, an assessment of need for treatment must be completed, and court hearings proceed.

Care orders are implemented in specially certified LVM and LVU homes, under the authority of the National Board for Institutional Care (SiS).

The number of adults undergoing compulsory care was 1,029 persons in 2003, whereof 301 were women, and the number of youths was 1073, whereof 373 were women [8].

### Subjects

A consecutive sample of 132 female drug abusers treated in compulsory care at the residential centre Lunden in Lund, Sweden, from 1997-01-01 to 2000-12-31 were selected. Women were included if they had been psychiatrically assessed at request of the local municipal authorities.

The women were followed over five years after treatment. The treatment unit is reserved for treatment of women exclusively, with a focus on women's issues and drug addiction. Subjects went through a formal diagnostic procedure at intake, covering somatic complaints, psychiatric and psychological problems, and background factors using standardized instruments. Patients were requested to give written consent to participate in the study, allowing the researchers to use data for research purposes and to retrieve register data. At follow-up, subjects were requested to confirm consent.

### Measures

At baseline assessment, subjects in the sample were assessed for psychiatric disorders (Axis I disorders assessed through SCID-I interview [9], and SCID-II interviews [10], were given a thorough medical examination, completed several personality tests, intelligence tests, and completed psychiatric self-rating scales. When indicated, women also underwent neuropsychological assessment.

The interview also included cross-sectional data, a Swedish adaptation of the Addiction Severity Index [11], standardized by a research team at the Swedish Board of Institutional Care (DOK) together with a longitudinal interview, an adapted version of the timeline follow-back interview covering the same seven problem areas and rating scales as in DOK. The Timeline Follow-Back [TLFB] interview is a structured format for assessing retrospective data that has been extensively validated [12]. The TLFB was conducted covering the entire period from discharge to follow-up (i.e., 60 months). Due to the long time covered, subjects were asked to indicate events in 6 month windows.

For the present study, we used only the part of the Timeline FollowBack Interview covering criminal charges and prison. We used four categories of legal problems: whether they had been charged with a drug offence, charged with theft, charged with violence, and whether they had been taken into an prison under the Swedish Prison and Probation Service.

The corresponding questions were: "have you been convicted of the following crimes: If yes, how many times?" The timeline then contained four categories in separate rows (drug related crimes, property crimes, violent crimes, and other crimes). Each column was then labelled with a year and letters indicating spring (V, for Swedish Vår), and fall (H for Swedish Høst). The years covered were 1997 to 2005.

Official records on all types of judicial sanctions were retrieved from The National Council for Crime Prevention (BRÅ), Stockholm, Sweden. The database has consistent penalty data on all persons having been in contact with the Swedish judicial system back to 1975 and up to the present day, and includes charges as well as sentences. We used data from the time of discharge for patients, and until the end of 2004. For each observation year, we recorded whether patients had been charged with a crime in one of the three categories, regardless of whether the charges lead to conviction or not.

### Follow-up procedure

At approximately 60 months post discharge, subjects were re-interviewed face-to-face by five independent clinical psychologists, and administered a battery of self-report questionnaires, partly the same as at index treatment admission.

The participants were traced trough social security and tax registers and information was added from significant others, staff in institutions, prisons and social bureaus, etc. At follow-up the diagnostic assessment was repeated and new areas added.

Since subjects were entered into the study over a 4-year period, the number of observations varied from year to year. The subjects who entered the study during 1999–2000 had some missing information from the database, as they were re-interviewed after 2004, and thus were interviewed about some years for which we did not have information from the criminal justice database.

### Statistical analyses

Comparisons between the two sources of information were made for each observation year, and for the whole period. That is, one data file was constructed containing one observation for each subject, with self-reported and file-recorded outcome in each area as variables. A second data-file was constructed with several observations for each woman, one for each year for which the woman had been interviewed. For example, if a woman had been discharged in 1998 and re-interviewed in 2003, she will have 6 observations in the data-file for the years 1998–2003. If a woman had been discharged in 2000 and reinterviewed in 2005, she will have 5 observations in the data-file for the years 2000–2004 (as we did not have complete data for the year 2005). This second data-file was used to estimate the agreement within each year, and to conduct the regression analyses. The information collected from the interviews and the official records were essentially identical: simple yes/no responses to the questions: "was Y charged with theft/drug related offences/violence in the year 200X?", or " was Y sentenced to prison in the year 200X?"

For each category of outcome, a Maximum Likelihood random effects logistic regression was conducted to assess whether self-reported criminal justice system involvement in a given category was related to observed crime in the register. In order to assess the specificity of the relations between recorded and self-reported criminal justice system involvement, we conducted these analyses controlling for other types of crime (i.e., to control for the fact that some women may indiscriminately have reported several types of charges, regardless of whether they had actually faced all types of charges). While logistic regression analyses are not commonly reported as indicators of convergent validity, using the random effects regression allows us to take advantage of the fact that the same women were observed in several different years, controlling for repeated measurements of the same persons.

We also calculated the κ for agreement, and the false negative and false positive rate for every observation year. The κ reliability coefficient can vary from -1 to 1. Values between 0.40 and 0.59 are considered fair agreement, those between 0.60 and 0.74 reflect good agreement, and those above 0.74 indicate excellent agreement [13]. The frequency of false negatives and false positives was calculated, with false positives measured as the proportion of inaccurate reporting of an offence out of all women without a record, and false negatives measured as the proportion of inaccurate offenders out of all women with a record. These frequencies were calculated for each year, and for the total follow-up period.

In order to compare the frequency of events for self-report and official record, Cochran's Q was used [14]. This comparison was made for ever reporting an event, that is, a comparison of the likelihood that subjects would report an event at any point during follow-up vs. the likelihood that an official record would be found. Finally, we estimated the independent associations between official records and self-reported events, controlling for other events, in a maximum likelihood random effects logistic regression.

## Results

### Sample description

The original sample consisted of 132 women treated in compulsory care. A total of 6 women refused to give consent to participate in the study (all at baseline). At follow-up, 6 had died. In total, 109 were interviewed, and of these, 106 were administered the timeline follow-back interview, and could thus be included. A total of 11 subjects were lost to follow-up. Thus, 84% of living subjects could be included. The drugs used were stimulants, mainly amphetamine (51%), opiates, mainly heroin (35%), alcohol (7%), or other drugs (all<3%). A total of 78% were diagnosed with personality disorders according to SCID-II interview, and 60% had an axis I disorder not related to substance use.

The mean number of observation years that could be linked was 4.1 per subject (range = 2–6). The reason that the number of observation years is slightly lower than the number of follow-up years is that data from the criminal justice database were only available up until 2004, and most subjects were interviewed later than 2004.

### Agreement between official records and self-reported criminal justice system involvement

The proportion of over-reporters and under-reporters and κ coefficients for the total period and for each year are shown in Table 1. Years are numbered, so that the year during which the interview was conducted is numbered 0, the previous year 1, and the year before that 2, etc. Results of ML random effects logistic regression are shown in Table 2.

**Table 1:** Data of agreement

**Year**	**N**	**% with record**	**% agreement**	**False negative rate**	**False positive rate**	**κ**
**Prison**						
Year of interview	43	7%	98	0	2	0.79
1 year before	105	8%	95	33	2	0.68
2 years before	105	7%	91	14	8	0.53
3 years before	87	10%	89	60	9	0.23
4 years before	61	7%	95	33	3	0.55
5 years before	38	11%	87	60	6	0.37
Ever	106	18%	85	37	10	0.51
**Drug related offences**						
Year of interview	43	0%	91	9	0	NA
1 year before	103	8%	84	85	9	0.36
2 years before	101	14%	83	36	14	0.42
3 years before	83	13%	75	55	21	0.18
4 years before	59	8%	71	80	24	0.00
5 years before	35	9%	91	50	3	0.52
Ever	108	39%	63	40	35	0.24
**Theft**						
Year of interview	41	10%	90	50	7	0.29
1 year before	102	11%	84	36	13	0.38
2 years before	99	7%	79	57	18	0.13
3 years before	85	14%	75	78	19	0.18
4 years before	61	13%	70	75	22	0.02
5 years before	36	8%	89	60	3	0.44
Ever	106	36%	70	34	28	0.37
**Violent**						
Year of interview	43	7%	93	100	0	NA
1 year before	104	6%	88	88	5	0.08
2 years before	104	6%	91	80	5	0.14
3 years before	86	7%	87	78	5	0.20
4 years before	60	7%	87	83	6	0.13
5 years before	37	11%	84	75	9	0.16
Ever	105	27%	74	71	9	0.23

**Notes: **Numbers represent time from interview to the time that the event took place (e.g., 0 is the same year as the interview, 1 is the previous year, 2 is two years before the interview, etc.). NA: Not applicable.

**Table 2:** Results of maximum likelihood random effects regression

	Model 1		Model 2	
	Odds ratio/Rho	Z	Odds ratio/Rho	Z

Prison				
Official record of prison	70.60	***5.58	47.26	***4.81
Official record of theft	NA		0.91	-0.15
Official record of drugs offences	NA		2.35	1.27
Official record of violence	NA		2.23	1.10
Theft				
Official record of theft	3.30	*2.34	1.96	1.31
Official record of drugs offences	NA		5.45	**3.35
Official record of violence	NA		1.28	0.40
Official record of prison	NA		4.80	*2.44
Drugs				
Official record of drugs offences	6.66	***3.76	5.17	**3.33
Official record of theft	NA		1.84	1.21
Official record of violence	NA		1.70	0.92
Official record of prison	NA		4.24	*2.65
Violence				
Official record of violence	6.28	*2.45	5.59	*2.00
Official record of drugs offences	NA		1.20	0.20
Official record of theft	NA		0.50	-0.82
Official record of prison	NA		2.49	0.92

**Notes: *Model 1***: Logistic regression with a random effect for intra-subject variation and the corresponding official record as predictor. ***Model 2: ***Logistic regression with a random effect for intra-subject variation and official record for all categories as predictors. † p < 0.10. * p < 0.05. ** p < 0.01 *** p < 0.001. **NA: **Not applicable as this effect is not included in present model.

### Drug offences

For ever being charged with drug related offences, the percent agreement was 63, and κ was 0.24 (p = 0.006). The false positive rate was 35%, and the false negative rate was 40%. Subjects were no more or less likely to report drug offences than were the official records (48% in records vs. 50% in self-report, Q(1) = 0.90, p = 0.34). False negative and false positive rates varied from year to year.

The random effects logistic ML regression showed that self-reported drug offences were significantly related to observed record in the criminal offences register (OR = 6.66 for presence of record in the presence of self-reported drug record, Z = 3.76,s p < 0.001). After controlling for record of other offences than theft and prison, recorded drug offences remained significantly associated with self-reported drugs offences (OR = 5.17, Z = 3.33, p < 0.01), records of theft and violence was unrelated to self-reported drugs offences, but record of prison was related to self-reported drugs offences (OR = 4.24, z = 2.65, p < 0.05).

### Theft

For ever being charged with theft, the percent agreement was 70%, and κ was 0.37 (p < 0.001). The false positive rate was 28, and the false negative rate was 34%. Subjects were no more or less likely to report theft than were the official records (42% vs. 36%, Q(1) = 1.12, p < 0.29).

The random effects logistic ML regression showed that self-reported theft was significantly related to observed record of theft in the criminal offences register (OR = 3.30 for self-report of theft in the presence of theft recorded, Z = 2.34, p < 0.05). After controlling for record of other offences and prison, self-reported theft was no longer significantly associated with a record of theft (OR = 1.96, Z = 1.31, NS). Self-reported theft was predicted by drugs offences (OR = 5.45, Z = 3.35, p < 0.01), and prison (OR = 4.80, Z = 2.44, p < 0.05).

### Violence

For ever being charged with violence, the percent agreement was 74%, and κ was 0.23 (p < 0.01). The false positive rate was 9%, and the false negative rate was 71%. Subjects were significantly less likely to report violence than were the official records (14% vs. 27%, Q(1) = 6.26, p < 0.013).

The random effects logistic ML regression showed that self-reported violence was significantly related to observed record of violence in the criminal offences register (OR = 6.28 for self-report of violence in the presence of violence recorded, Z = 2.45, p < 0.001). After controlling for record of other offences and prison, self-reported violence remained significantly associated with a record of violence (OR = 5.59, Z = 2.00, p < 0.05).

### Prison

For ever being in prison during the follow-up period, the percent agreement was 85%, and κ was 0.51 (p < 0.001). Subjects were not significantly more or less likely to report prison than were the official records (18% vs. 20%, Q(1) = 0.25, p < 0.62).

The random effects logistic ML regression showed that self-reported prison was significantly related to observed record of prison in the criminal offences register (OR = 70.60 for presence of prison record in the presence of self-reported prison, Z = 5.58, p < 0.001).

After controlling for record of specific offences, recorded prison sentence remained associated with self-reported prison (OR = 47.26, Z = 4.81, p < 0.001).

### The possible effects of memory

To test the influence of memory, we divided the time period up into two categories: year 0–3 and year 4–7. Within each time period, we analyzed agreement between self-reported criminal justice system involvement and criminal justice records. For drug related offences, the agreement for more recent events (past three years) was 0.48, and for earlier events it was 0.23. For theft the agreement for recent events was 0.32, and for more distant events, the agreement was 0.32. For violence, the agreement for more recent events was 0.09, and for earlier events was 0.31. And finally for prison, the agreement for more recent events was 0.53, and for earlier events was 0.38. Thus, the picture does not consistently show that failure to recall events that occurred several years ago is the main source of disagreement for these events, although for drug related events and prison, the findings favoured more recent events. For theft, no difference was found, and for violence the pattern was in the opposite direction.

## Discussion

This study is one of rather few to assess the validity of criminal behaviour in a follow-up study with a high-quality database of criminal records with national coverage. Compared with most other studies, the present one differs by testing statistically whether agreement is better than chance, rather than simply reporting the percent agreement, and by conducting formal statistical tests of whether patients under- or over-report. Percent agreement, as used in earlier studies, over-estimates agreement, because it fails to take into account whether bias is present, and that extreme base-rates may give high percentage agreement, even with no actual correspondence (e.g., if both have a base-rate of 5% for an event and no correspondence exists, percent agreement will be 90).

A strength of this study is that the sample was well defined, and that follow-up rate was high for such a long follow-up period with data available for 84% of living subjects.

The overall proportion of subjects who would be classified as having been charged with a given crime differed very little between data from the criminal justice database and self-reported events. False negatives and false positives cancelled out each other for three of four categories, with the exception of violence; some indication of bias was present for violence, which was reported significantly more often in the records than by subjects.

Agreement between self-reported criminal behaviour and criminal justice records was fair at best in the present sample. For specific categories of outcome (violence, theft and drug related offences) and prison, κ values were generally in the poor-fair range in any given year, and even when requiring only agreement about any event within the total observation time, agreement was fair for two of four categories, theft and prison, and poor for two, violence and drugs offences. Thus, the classification of a given person as falling within a category or not in a given year is done with some uncertainty, although far better than would be expected by chance. Also, controlling for other types of legal outcomes, three of four self-reported outcomes remained significantly associated with the corresponding record.

Several sources can contribute to variability in the agreement between self-reported criminal justice system involvement and official sources. Shame, stigma, and social desirability issues may differ between types of criminal events, and the perceived significance of an event may contribute to remembering it. For example, being sentenced to prison leads to a long series of events that are easy to remember, whereas being charged and receiving a fine may be a much less significant result. This could explain why the best agreement was found for prison sentences. Women reported violence significantly less common than violence was recorded in the official records, which may indicate violence is particularly shameful for women, since they do not report or recall these events.

While memory may play a role, we did not find that failure to recall events that were further away in a more distant past was consistently more common than failure to recall more recent events.

If research studies address the overall amount of crime committed by a population in a given period, the modest agreement between sources of information may be of relatively little concern. However, if specific predictors of legal outcomes are studied, including the impact of specific treatment interventions, uncertainty as to who actually did what when may be a serious problem.

Some limitations must be acknowledged for the present study. The study included only women, and therefore the findings may not generalize to studies of male substance abusers. Also, women from compulsory care may differ from other substance abusers in ways that make them respond differently to answers about criminal involvement. Also, the relatively low base-rate of all types of criminal behaviour within a given year limits reliability. However, when using the whole period of five years follow-up, base-rates were not very low, even for violence with 27% being charged with violence at some point during the period.

Another limitation is that the comparison could only cover crimes that had come to police knowledge. This means that the study is related only to the validity of self-reported criminal justice system involvement, and may not reflect the validity of self-reported criminal behaviour. A proportion of crimes never lead to charges, and an unknown proportion of crimes are not even reported to the police. Thus, the findings of the present study are relevant only for self-reported charges and incarceration, not for crimes that have never lead to charges.

Also, whether or not the women actually committed the crimes they were charged with goes beyond the scope of the present study, although we recognize it as an interesting question.

Finally, while prostitution is a common source of income for female substance abusers that could be of interest, doing sex for money is not illegal in Sweden (although paying money for sex is illegal), and thus cases concerning prostitution would not be recorded under the women's person register numbers. Thus, results might differ from countries where prostitution is an offence.

## Conclusion

In conclusion, the present study suggests that self-report in a timeline follow-back interview is a fairly valid measure of criminal justice system involvement in a follow-up context. Clearly, there was a significant relationship between self-reported charges and incarceration, and events in the record, and the logistic regression analyses showed that there was also some specificity for the specific types of offences included in the study.

## Competing interests

The author(s) declare that they have no competing interests.

## Authors' contributions

IJ and MF conceived of the follow-up study, and designed and planned data collection. IJ planned and overlooked data collection at baseline, and MF overlooked and planned data collection at follow-up. MH suggested the present analyses and carried out the statistical analyses. All authors read and approved the final manuscript.

## Pre-publication history

The pre-publication history for this paper can be accessed here:


